# From exchangeability to rational belief: a cognitive interpretation of de Finetti’s theorem

**DOI:** 10.3389/fpsyg.2025.1621552

**Published:** 2025-12-02

**Authors:** Tommaso Costa

**Affiliations:** 1 GCS-fMRI, Koelliker Hospital and Department of Psychology, University of Turin, Turin, Italy; 2 FOCUS Laboratory, Department of Psychology, University of Turin, Turin, Italy; 3 Neuroscience Institute of Turin (NIT), Turin, Italy

**Keywords:** probabilistic reasoning, de Finetti’s representation theorem, rational inference, uncertainty, E. T. Jaynes, logic of belief updating, symmetry, exchangeable sequences

## Abstract

Probabilistic reasoning is central to many theories of human cognition, yet its foundations are often presented through abstract mathematical formalisms disconnected from the logic of belief and learning. In this article, we propose a reinterpretation of de Finetti’s representation theorem as a principle of rational inference under uncertainty. Building on the framework developed by E. T. Jaynes—where probability is viewed as an extension of logic—we show that the structure of de Finetti’s theorem mirrors the logic of belief updating constrained by symmetry. Exchangeable sequences, which treat observations as order-invariant, lead naturally to a representation in which probabilities are weighted averages over latent causes. This structure is formally analogous to the role of partition functions in statistical models, where uncertainty is distributed across hypotheses according to constraints and prior expectations. We argue that this correspondence is not merely mathematical but reveals a deeper cognitive interpretation: the mind, when faced with symmetry and incomplete information, may infer in ways that implicitly reflect maximum entropy principles. We illustrate this connection with a simple example and discuss how the underlying structure of de Finetti’s theorem can inform our understanding of inductive learning, probabilistic belief, and the rational architecture of cognition.

## 1 Introduction: from statistical symmetry to cognitive rationality

Probabilistic reasoning is widely recognized as a hallmark of human cognition. Whether we are making everyday judgments under uncertainty or evaluating scientific evidence, we routinely update our beliefs in response to data. In recent decades, Bayesian models have gained prominence as both normative and descriptive accounts of this inferential process (Griffiths et al., 2008; Oaksford and Chater, 2007). However, many foundational aspects of these models remain underexplored within psychological theory—particularly regarding the rationale behind certain probabilistic structures and how they might reflect deeper cognitive principles.

One key foundational concept is exchangeability: the idea that the order in which observations are received should not influence our beliefs if we have no reason to believe that the order carries meaningful information. This symmetry condition plays a central role in Bruno de Finetti’s representation theorem—a result that shows how any sequence of exchangeable observations can be treated as if it were generated by a latent, but unknown, probability distribution (de Finetti, 1990). In other words, assuming exchangeability is tantamount to assuming that the data are conditionally independent and identically distributed, given some hidden parameters. By symmetry, we mean invariance under permutations of the data indices: the joint probability remains unchanged if the order of observations is permuted. Intuitively, no observation should be privileged if order carries no meaning. We use the mind to denote the abstract reasoning system and the brain to refer to the biological substrate.

The notion of exchangeability has already played a foundational role in psychological models of learning and categorization. Early formulations of the *Rational Model of Categorization* (Anderson, 1991) and subsequent *nonparametric Bayesian models* (Sanborn et al., 2006; Griffiths et al., 2007, 2011; Gershman and Blei, 2012) all rely on exchangeability assumptions to explain how learners generalize from limited data. Our contribution does not aim to introduce this idea to psychology but rather to reinterpret de Finetti’s theorem as a *cognitive principle* of rational belief formation under symmetry—an interpretation that unifies these prior developments within a logic-of-belief framework inspired by Jaynes.

Although this theorem is often treated as a technical result in mathematical statistics, it has profound implications for cognitive science. It provides a formal justification for inductive reasoning under minimal assumptions and suggests that belief formation, when guided by symmetry, naturally leads to probabilistic models that integrate over uncertainty about latent causes (Zabell, 1988).

In this article, we revisit de Finetti’s theorem not as a piece of abstract mathematics but as a cognitive principle of rational belief formation. Drawing inspiration from E. T. Jaynes’ view of probability as an extension of logic (Jaynes, 2003), we argue that the structure of de Finetti’s representation mirrors a more general pattern in human inference: beliefs are shaped not only by data and prior expectations but also by structural constraints such as symmetry and invariance. We show that the integral form of de Finetti’s theorem is conceptually analogous to the partition function in statistical models—a mathematical device that aggregates plausibility across configurations under constraint (Caticha, 2012).

We contend that this analogy is not merely formal. It highlights a broader view of inference as the product of structured ignorance: when we lack specific information, the rational strategy is to distribute belief in a manner that respects the known constraints without introducing unjustified assumptions. We suggest that this logic, rooted in symmetry and entropy, may reflect core aspects of how the human mind handles uncertainty.

The article proceeds as follows: In Section 2, we explore the notion of exchangeability and its role in structuring probabilistic beliefs. Section 3 introduces de Finetti’s representation theorem and discusses its implications for latent-variable inference. In Section 4, we develop the conceptual analogy between this theorem and partition functions. Section 5 presents a simple worked example, and Section 6 discusses the broader implications for cognitive modeling, learning, and the architecture of rational inference.

## 2 Exchangeability and the logic of belief

In many real-world situations, we form beliefs about uncertain events without knowing their generative mechanisms. When direct causal information is unavailable, we often rely on structural assumptions—such as symmetry—to constrain our inferences. One of the most powerful and general such assumptions is exchangeability: the idea that the joint plausibility of a sequence of observations does not depend on the order in which they are received.

Originally introduced by de Finetti (1990), exchangeability provides a minimal yet meaningful constraint on belief formation. It states that for any finite sequence of random variables X₁, X₂, …, Xₙ, the joint probability assignment should remain invariant under any permutation of the indices:


$$P(X^{1}=x^{1},\ldots ,\mathrm{Xn}=x_{n})=P(X_{\left\{\pi (1)\right\}}=x^{1},\ldots ,X_{\left\{\pi (n)\right\}}=x_{n})$$

For any permutation π. In cognitive terms, this means that no observation is treated as privileged simply due to its position in the sequence. If the data are indistinguishable in their evidential value, our beliefs should reflect that symmetry.

Exchangeability can be regarded as a rational default assumption in the absence of temporal or causal structure. This concept implies that our beliefs about future observations should be shaped by the frequency of past observations, rather than their order. In fact, as de Finetti’s representation theorem shows, assuming exchangeability leads to the conclusion that the data can be modeled as conditionally i.i.d. (independent and identically distributed) given some latent variable *θ*. In psychological terms, it is as if the mind implicitly posits an underlying generative mechanism, without committing to a specific causal model. Exchangeability is both liberating—because it removes arbitrary assumptions about order—and constraining, since it forces us to treat observations symmetrically, imposing coherence on belief states.

This perspective aligns with a broader understanding of human cognition as model-based and generative. The mind does not merely register frequencies—it constructs abstract representations that support prediction and generalization (Tenenbaum et al., 2006). Exchangeability provides the structural foundation for this generative stance, allowing the learner to group observations by statistical type rather than by sequence.

Moreover, the assumption of exchangeability aligns naturally with Bayesian approaches to cognition, where beliefs are updated in light of evidence via conditional probabilities. By enforcing symmetry over the data, exchangeability narrows the space of rational belief states. It ensures that posterior distributions are coherent with respect to plausible ignorance about ordering—an epistemic constraint that mirrors the way people often reason when they lack contextual or temporal cues (Zabell, 1988).

Taken together, these considerations support the idea that exchangeability is not merely a technical device but a psychologically plausible principle for organizing uncertain information. In the next section, we show how this symmetry gives rise to a specific representation of belief: the de Finetti integral, which describes the probability of the data as an average over latent causes weighted by prior plausibility.

Nevertheless, exchangeability is a conditionally rational assumption—it applies only in environments that truly exhibit symmetry with respect to the observed data. In natural settings, temporal order, causal dependencies, or contextual information often break exchangeability. Human reasoning appears to adaptively toggle between symmetric and asymmetric models, depending on whether such structures are perceived as informative (Griffiths and Tenenbaum, 2006). Therefore, while exchangeability provides a coherent normative baseline for reasoning under ignorance, real-world inference frequently departs from it when temporal or causal cues justifiably constrain belief. Making these boundary conditions explicit helps reconcile the cognitive interpretation of exchangeability with evidence for context-sensitive and causally informed reasoning.

## 3 The de Finetti’s representation theorem and structured uncertainty

The representation theorem that bears de Finetti’s name is often introduced as a foundational result in Bayesian statistics, but its cognitive implications are equally significant. The theorem states that any infinite exchangeable sequence of binary random variables can be represented as a mixture of i.i.d. processes. In formal terms, if a sequence $X^{1},X^{2},\ldots$ is exchangeable, then there exists a probability measure *μ*(*θ*) such that:


$$P(X^{1}=x^{1},\ldots ,\mathrm{Xn}=x_{n})=\int_{0}^{1}\;\theta^{s_{n}}{\left(1-\theta \right)}^{\left\{n-s_{n}\right\}}\;\mathrm{d\mu}(\theta )$$

Where sₙ is the number of “successes.” Under exchangeability, we can treat the data as if they were generated by a fixed but unknown parameter θ, drawn from a prior distribution μ. Cognitively, this suggests that a rational agent implicitly reasons over uncertain latent causes (Zabell, 1989).

The psychological interpretation is profound: exchangeability leads to structured uncertainty, where beliefs are shaped by both data and latent generative assumptions. The prior μ(θ) expresses subjective uncertainty about the underlying process, while the likelihood reflects how well each θ explains the data. The integral yields a belief-weighted average over these hypotheses.

This aligns with the view that the mind constructs abstract models even when information is incomplete (Griffiths and Tenenbaum, 2006). The brain may not compute integrals explicitly, but it approximates such reasoning through sampling, neural coding, or analogical inference.

Crucially, the prior *μ*(*θ*) is not arbitrary. Under pure exchangeability, one rational choice is the uniform prior, which maximizes entropy under the assumption of no further constraints (Jaynes, 2003). However, the maximum entropy principle is always problem-specific: its solution depends on the stated constraints and the measure defined over the parameter space. A reparameterization—for instance, from probability to odds or log-odds—can change the base measure and yield different maximum-entropy priors. Alternative principled defaults, such as Jeffreys’ prior or reference priors (Bernardo, 1979; Jeffreys, 1946), offer invariant formulations of ignorance that remain consistent across parameterizations. Mentioning these points clarifies that “ignorance” does not uniquely select a uniform prior, but instead motivates a class of priors satisfying structural neutrality. This reinforces the idea that symmetry implies indifference: without distinguishing evidence, all possibilities are treated equally. Maximum entropy applies to the prior: with only exchangeability, the uniform prior maximizes entropy. In contrast, posteriors reduce entropy as data accumulate.

Psychologically, when people face symmetric conditions (e.g., judging outcomes with no contextual cues), they may default to belief structures resembling maximum entropy reasoning. Empirical evidence indicates that people often behave as if they are averaging over hidden causes under uncertainty (Zhu et al., 2020). De Finetti’s theorem provides a normative justification for this behavior.

In the next section, we draw an analogy between de Finetti’s representation theorem and the partition function—a statistical model concept that distributes plausibility over constrained hypotheses.

## 4 From physical to epistemic partition functions

The core property of exchangeable sequences is their *latent-*var*iable representation*: by de Finetti’s theorem, any order-invariant probability distribution can be expressed as a mixture over hidden causes. The analogy with the partition function is not intended as a new property or corollary but as a complementary formal perspective on the same structure—highlighting that integrating over latent causes plays a role mathematically similar to normalization in physical systems. However, both involve integrating over a space of latent possibilities, weighting each by plausibility, and producing a normalized distribution constrained by the system’s structure.

In physics, the partition function Z aggregates the contributions of all microstates, often expressed as $Z=\int e^{\left\{-\mathrm{\beta E}(\theta )\right\}}\rho (\theta )\;\mathrm{d\theta}$, where E(θ) is energy and ρ(θ) is the density of states. This function normalizes the Boltzmann distribution and reflects how probability is allocated under energetic constraints.

In Bayesian inference, de Finetti’s representation theorem similarly integrates over a latent parameter space: P(D)=∫p(D∣θ)dμ(θ). Here, the likelihood is analogous to energy, and the prior resembles the density of states. The integral serves as a Bayesian partition function (MacKay, 2003).

This analogy suggests that human inference under uncertainty can be understood in terms of epistemic constraints. Therefore, the latent-variable interpretation remains primary; the concept of an “epistemic partition function” merely rephrases the same marginalization principle in energetic or normalization terms. Just as physical systems settle into distributions based on energy and temperature, rational agents form belief states that balance evidence (likelihood) and prior constraints. By ‘epistemic partition function,’ we refer to the Bayesian marginal likelihood, that is, which is the integral of the likelihood weighted by the prior.

Seen in this light, de Finetti’s integral is not merely technical—it expresses how structured ignorance is resolved. When generative mechanisms are unknown but the data are exchangeable, beliefs are distributed across hypotheses in a principled, constrained fashion.

Therefore, the epistemic partition function reflects total plausibility assigned to the observed data across latent models. It plays a central role in Bayesian model selection and cognitive evaluation (Spiegelhalter et al., 2002). It highlights that beliefs are shaped not only by evidence but also by the structure of the reasoning space itself.

More fundamentally, the existence of a partition function indicates that any exchangeable sequence implicitly assumes a latent generative structure. De Finetti’s theorem guarantees that order-invariant observations can always be represented as independent samples from a latent distribution, making the inference over this latent space the true cognitive task. This perspective emphasizes that rational inference is not only about belief updating but also about discovering the hidden structure that explains why exchangeability holds in the first place. Therefore, the “partition function” is not merely a normalization term but a formal bridge between symmetry at the observational level and causality at the representational level.

Conceptually, describing the marginal likelihood as an epistemic partition function offers more than a mere change in terminology. It makes explicit that rational belief updating can be understood as an energy-balancing process: the mind distributes plausibility across hypotheses to minimize epistemic “free energy,” trading off evidence fit against prior constraints. This perspective links Bayesian inference to potential neurocomputational mechanisms, such as those proposed in predictive coding and free-energy models of brain function (Friston, 2010). It also suggests testable predictions: when the symmetry of the hypothesis space or the entropy of prior constraints is experimentally manipulated, belief updating should show measurable shifts in confidence and variability. Therefore, the partition function framing not only restates Bayesian integration but also extends it into a framework that connects epistemic structure, computational efficiency, and cognitive phenomenology.

In the next section, we illustrate this process through a simple example of exchangeable inference.

## 5 A simple example of exchangeable inference

To illustrate the structure of exchangeable inference, a simple binary scenario was considered. Suppose an agent observes 10 events, 7 of which are classified as “successes.” Assuming exchangeability and no additional knowledge, the rational prior over θ∈[0,1] is uniform.

**Figure 1:**
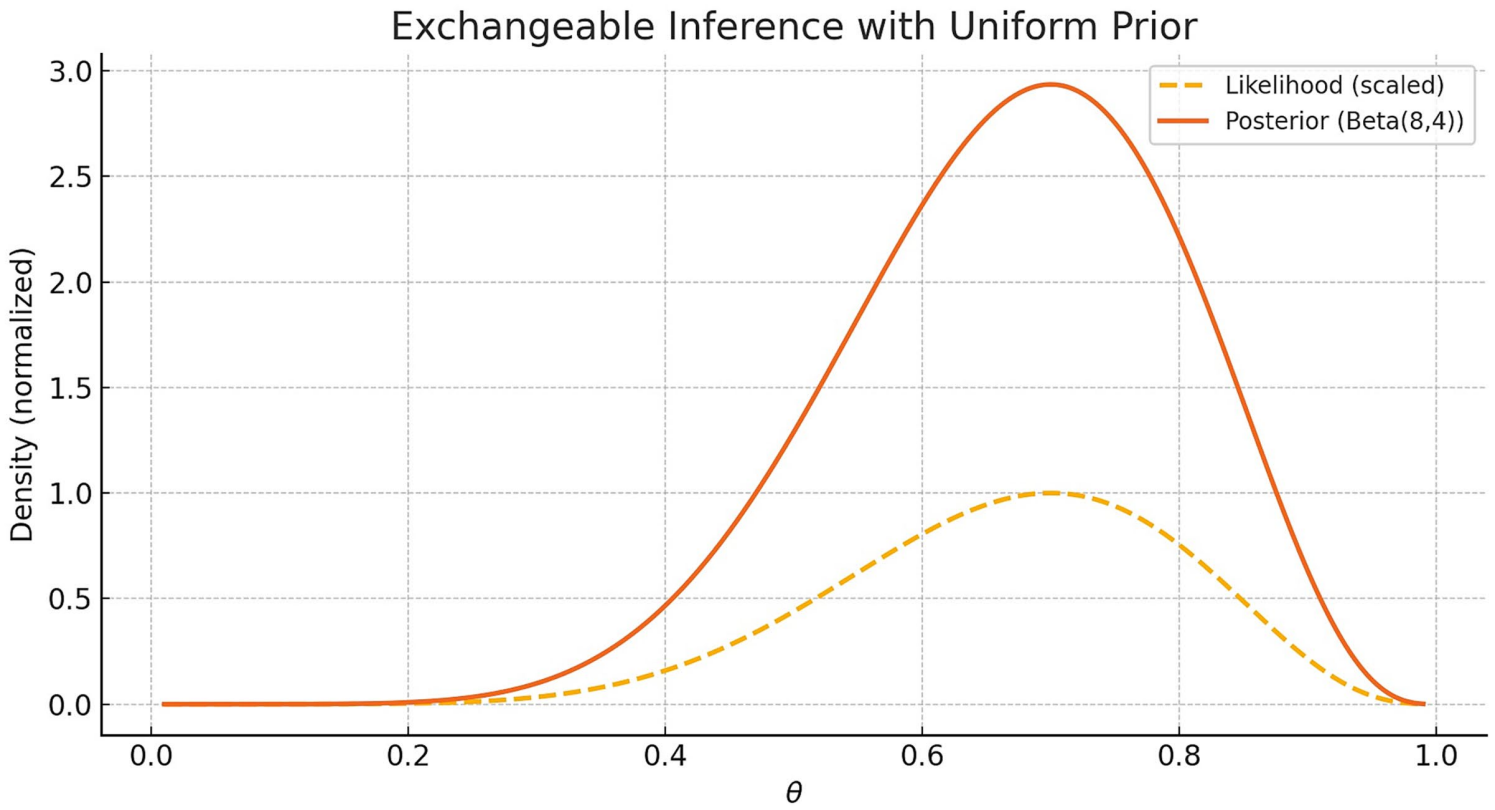
Posterior distribution over the latent success probability θ after observing seven successes and three failures out of 10 binary events. The agent assumes exchangeability and starts with a uniform prior. The dashed line shows the unnormalized likelihood function $\theta^{7}{\left(1-\theta \right)}^{3}$, reflecting the fit of each hypothesis to the data. The solid line shows the resulting posterior distribution, which corresponds to a Beta (8, 4) distribution. This illustrates how rational inference, under symmetry and minimal assumptions, leads to a coherent belief distribution over latent causes.

The likelihood of the data is proportional to $\theta^{7}{\left(1-\theta \right)}^{3}$. The posterior, by Bayes’ rule, is a Beta (8, 4) distribution, combining the prior and data. Figure 1 shows the scaled likelihood and the resulting posterior.

This posterior reflects updated beliefs, shaped by both the observed frequency and the structural constraint of symmetry. No causal model is assumed; inference emerges from minimal assumptions.

Cognitively, this illustrates that coherent belief updating can arise from symmetry and data alone. The agent does not need to know the true process—by treating the data as structurally identical, a rational posterior is formed through integration over latent possibilities.

This supports the study’s research hypothesis: exchangeability allows rational inference under ignorance. The posterior is not arbitrary—it reflects a principled aggregation of evidence and uncertainty. Rational inference arises from the Bayesian framework, with de Finetti’s theorem serving as one case. Partition exchangeability (Amiryousefi et al., 2022) is another route to rational inference.

## 6 Implications for cognitive science and psychology

This reinterpretation of de Finetti’s theorem carries several implications for cognitive science. First, it formalizes how beliefs can be updated without detailed causal knowledge. Symmetry-based assumptions constrain inference and support generalization under uncertainty (Tenenbaum et al., 2011).

Second, linking the de Finetti integral to the partition function offers a unified account of belief integration and latent-structure discovery. It formalizes how the mind aggregates evidence across hypotheses while implicitly learning the hidden parameters that generate exchangeable data. This view directly connects with nonparametric Bayesian models of categorization and latent-cause inference (Anderson, 1991; Gershman and Niv, 2010), in which learners infer not only the probabilities of outcomes but also the structure that generates them.

Third, this explains default reasoning. People often treat outcomes as equiprobable without evidence to the contrary (Xu and Garcia, 2008). This reflects maximum entropy reasoning under symmetry, as prescribed by de Finetti.

Fourth, this framework contributes to ongoing discussions of bounded rationality. While human heuristics may deviate from exact Bayesian inference, they can be interpreted as approximate implementations of symmetry-based inference under resource constraints. This interpretation resonates with rational process models (Sanborn et al., 2010) and supports the view that coherence, rather than computational precision, defines rational cognition. This perspective also aligns with extensive research showing that human judgment often relies on heuristics when information is limited or costly to process (Tversky and Kahneman, 1974; Gigerenzer and Gaissmaier, 2011).

Interestingly, Sanborn et al. (2010) showed that when Bayesian inference is implemented through sequential or sampling-based algorithms, apparent *order effects* can emerge even under an exchangeable generative model. These effects arise from algorithmic approximations rather than from violations of the underlying symmetry, reinforcing the idea that bounded rationality may reflect computational constraints on otherwise coherent probabilistic reasoning.

Finally, the framework invites empirical testing: do people behave as if they assume exchangeability? How do priors and symmetry affect inference? Investigating these questions can help connect probabilistic theory with real cognitive processes. At the physiological level, the brain approximates probabilistic averaging through parallel neural networks, integrating signals across billions of neurons and trillions of synapses.

Beyond infant statistical learning, several studies have examined exchangeability-like reasoning in adults. Research on adult statistical learning and generalization (Fiser and Aslin, 2002; Griffiths et al., 2010) indicates that people often assume order-invariant structures when evidence does not suggest causal or temporal asymmetry. At the same time, violations of exchangeability emerge when participants infer latent causes or temporal dependencies (Kemp et al., 2007; Gershman et al., 2010). These findings align with hierarchical Bayesian models of perception and belief updating, such as predictive coding (Friston, 2010) and active inference (Parr and Friston, 2017), in which the brain dynamically balances exchangeable and non-exchangeable structures depending on contextual cues. Incorporating these frameworks helps situate the proposed interpretation of de Finetti’s theorem within current computational accounts of cognition and neural inference.

Beyond cognitive and perceptual domains, recent research shows that human reasoning departs from exchangeability in contexts rich in moral or emotional content. When moral values, affective salience, or social identity are at stake, symmetry assumptions break down: people systematically privilege certain outcomes or agents, violating order invariance and probabilistic neutrality (Lloyd et al., 2023). These findings highlight that exchangeability is not only constrained by temporal or causal cues but also by motivational and moral factors that shape the perceived relevance of observations. Recognizing these departures helps delimit the ecological validity of exchangeability and clarifies that rational inference is context-dependent and sensitive to the cognitive and affective structure of the environment.

In addition to its foundational role, exchangeability underlies a broad class of cognitive models that implement rational inference through Bayesian nonparametric methods. The rational model of categorization (Anderson, 1991) can be derived from exchangeability assumptions, leading to a Dirichlet process prior over category partitions (Sanborn et al., 2006). Subsequent research unified this approach with hierarchical extensions (Griffiths et al., 2007, 2011) and explored how rational learners approximate such inferences using psychologically plausible algorithms (Sanborn et al., 2010). Parallel developments in latent-cause models (Gershman et al., 2010; Gershman and Niv, 2010; Gershman et al., 2015) and latent-feature representations (Austerweil and Griffiths, 2013) further demonstrate how exchangeability provides the mathematical backbone for flexible inductive generalization. Reviews by Gershman and Blei (2012) and Austerweil et al. (2015) summarize this research program, showing that exchangeability—when combined with hierarchical and nonparametric priors—supports adaptive inference across categorization, causal reasoning, and memory.

Finally, the assumption of full exchangeability has been generalized in richer frameworks of *partial exchangeability*, which relax symmetry constraints while preserving coherent probabilistic inference. This extension underlies models such as latent Dirichlet allocation (Blei et al., 2003), their psychological counterparts in topic learning (Griffiths et al., 2007), and recent connections between de Finetti’s theorem and large language models (Zhang et al., 2023; Ye and Namkoong 2024). These developments illustrate that exchangeability remains a central organizing principle even in the age of hierarchical, neural, and in-context Bayesian inference.

## 7 Conclusion: rational inference as structured ignorance

At its core, this article argues that rational belief does not require detailed generative models; however, it does require coherence under uncertainty. Exchangeability offers a principled basis for inductive inference when information is minimal. De Finetti’s theorem shows how beliefs can reflect structured ignorance—a plausible distribution across hypotheses guided by prior plausibility and observed data.

This is conceptually analogous to the partition function: a normalizing factor aggregating plausibility across constraints. More broadly, it offers a window into the logic of belief itself.

From this perspective, inference is not simply numerical—it is epistemic. It reconciles what is known with what is unknown, guided by symmetry and constraint. This aligns with traditions that view probability as an extension of logic (Jaynes, 2003; Carnap, 1950) and with a conception of the rational mind as sensitive to structural coherence.

Understanding inference as structured ignorance bridges cognitive science and probability theory. It unifies symmetry, plausibility, and uncertainty into a cohesive framework for rational belief. While full exchangeability provides the idealized foundation for rational inference under symmetry, many natural environments exhibit only *partial exchangeability*—that is, invariance within, but not across, contextual or hierarchical groupings. Extending the present framework to such cases connects de Finetti’s logic of symmetry with hierarchical Bayesian cognition, where rational belief remains coherent but is structured by context-dependent constraints. De Finetti’s theorem is pivotal but not unique. Partition exchangeability and other Bayesian formalisms also support inductive inference, with Bayesian logic as the overarching principle.

## Data Availability

The original contributions presented in the study are included in the article/supplementary material, further inquiries can be directed to the corresponding author.

## References

[ref1] Amiryousefi A. Kinnula V. Tang J. (2022). Bayes in wonderland! Predictive supervised classification inference hits unpredictability. Mathematics 10:828. doi: 10.3390/math10050828

[ref2] Anderson J. R. (1991). The adaptive nature of human categorization. Psychol. Rev. 98, 409–429. doi: 10.1037/0033-295X.98.3.409

[ref3] Austerweil J. L. Gershman S. J. Tenenbaum J. B. Griffiths T. L. (2015). “Structure and flexibility in Bayesian models of cognition” in Oxford handbook of computational and mathematical psychology (Oxford, UK: Oxford University Press), 187–208.

[ref4] Austerweil J. L. Griffiths T. L. (2013). A nonparametric Bayesian framework for constructing flexible feature representations. Psychol. Rev. 120, 817–851. doi: 10.1037/a0034194, PMID: 24219850

[ref5] Bernardo J. M. (1979). Reference posterior distributions for Bayesian inference. J. R. Stat. Soc. Ser. B 41, 113–147.

[ref6] Blei D. M. Ng A. Y. Jordan M. I. (2003). Latent Dirichlet allocation. J. Mach. Learn. Res. 3, 993–1022.

[ref7] Carnap R. (1950). Logical foundations of probability. Chicago, IL: University of Chicago Press.

[ref8] Caticha A. (2012). Entropic inference and the foundations of physics. Sao Paulo, Brazil: Brazilian Chapter of the International Society for Bayesian Analysis-ISBrA.

[ref9] de Finetti B. (1990). Theory of probability: A critical introductory treatment (Vols. 1–2, Machì A. Smith A., New York, NY: John Wiley & Sons.

[ref10] Fiser J. Aslin R. N. (2002). Statistical learning of higher-order temporal structure from visual shape sequences. J. Exp. Psychol. Learn. Mem. Cogn. 28, 458–467. doi: 10.1037//0278-7393.28.3.458, PMID: 12018498

[ref11] Friston K. (2010). The free-energy principle: a unified brain theory? Nat. Rev. Neurosci. 11, 127–138. doi: 10.1038/nrn2787, PMID: 20068583

[ref12] Gershman S. J. Blei D. M. (2012). A tutorial on Bayesian nonparametric models. J. Math. Psychol. 56, 1–12. doi: 10.1016/j.jmp.2011.08.004

[ref13] Gershman S. J. Blei D. M. Niv Y. (2010). Context, learning, and extinction. Psychol. Rev. 117, 197–209. doi: 10.1037/a0017808, PMID: 20063968

[ref14] Gershman S. J. Niv Y. (2010). Learning latent structure: carving nature at its joints. Curr. Opin. Neurobiol. 20, 251–256. doi: 10.1016/j.conb.2010.02.008, PMID: 20227271 PMC2862793

[ref15] Gershman S. J. Norman K. A. Niv Y. (2015). Discovering latent causes in reinforcement learning. Curr. Opin. Behav. Sci. 5, 43–50. doi: 10.1016/j.cobeha.2015.07.007

[ref16] Gigerenzer G. Gaissmaier W. (2011). Heuristic decision making. Annu. Rev. Psychol. 62, 451–482. doi: 10.1146/annurev-psych-120709-145346, PMID: 21126183

[ref17] Griffiths T. L. Canini K. R. Sanborn A. N. Navarro D. J. (2007). “Unifying rational models of categorization via the hierarchical Dirichlet process” in Proceedings of the annual meeting of the cognitive science society, Austin, TX: Cognitive Science Society. vol. 29.

[ref18] Griffiths T. L. Chater N. Kemp C. Perfors A. Tenenbaum J. B. (2010). Probabilistic models of cognition: exploring representations and inductive biases. Trends Cogn. Sci. 14, 357–364. doi: 10.1016/j.tics.2010.05.004, PMID: 20576465

[ref19] Griffiths T. L. Kemp C. Tenenbaum J. B. (2008). “Bayesian models of cognition” in The Cambridge handbook of computational psychology. ed. Sun R. (Cambridge University Press), 59–100.

[ref20] Griffiths T. L. Sanborn A. N. Canini K. R. Navarro D. J. Tenenbaum J. B. (2011). “Nonparametric Bayesian models of categorization” in Formal approaches in categorization. eds. Pothos E. M. Wills A. J. (Cambridge, UK: Cambridge University Press), 173–198.

[ref21] Griffiths T. L. Steyvers M. Tenenbaum J. B. (2007). Topics in semantic representation. Psychol. Rev. 114, 211–244. doi: 10.1037/0033-295X.114.2.211, PMID: 17500626

[ref22] Griffiths T. L. Tenenbaum J. B. (2006). Optimal predictions in everyday cognition. Psychol. Sci. 17, 767–773. doi: 10.1111/j.1467-9280.2006.01780.x, PMID: 16984293

[ref23] Jaynes E. T. (2003). Probability theory: The logic of science. (ed.) Bretthorst G. L. Cambridge, UK: Cambridge University Press.

[ref24] Jeffreys H. (1946). An invariant form for the prior probability in estimation problems. Proc. R. Soc. Lond. Ser. A Math. Phys. Sci. 186, 453–461. doi: 10.1098/rspa.1946.0056, PMID: 20998741

[ref25] Kemp C. Goodman N. D. Tenenbaum J. B. (2007). Learning and using relational theories. Adv. Neural Inf. Proc. Syst. 19, 753–760.

[ref26] Lloyd E. P. Starmans C. Friedman O. (2023). Moral reasoning is not probabilistic. Philos. Psychol. 36, 1004–1027.

[ref27] MacKay D. J. C. (2003). Information theory, inference, and learning algorithms: Cambridge, UK: Cambridge University Press.

[ref28] Oaksford M. Chater N. (2007). Bayesian rationality: The probabilistic approach to human reasoning. Oxford, UK: Oxford University Press. 10.1017/S0140525X09000284 19210833

[ref29] Parr T. Friston K. J. (2017). The active construction of the visual world. Neuropsychologia 104, 92–101. doi: 10.1016/j.neuropsychologia.2017.08.003, PMID: 28782543 PMC5637165

[ref30] Sanborn A. N. Griffiths T. L. Navarro D. J. (2006). A more rational model of categorization. In Proceedings of the 28th Annual Conference of the Cognitive Science Society. Vancouver, Canada: Cognitive Science Society. (pp. 726–731)

[ref31] Sanborn A. N. Griffiths T. L. Navarro D. J. (2010). Rational approximations to rational models: alternative algorithms for category learning. Psychol. Rev. 117, 1144–1167. doi: 10.1037/a0020511, PMID: 21038975

[ref32] Spiegelhalter D. J. Best N. G. Carlin B. P. van der Linde A. (2002). Bayesian measures of model complexity and fit. J. R. Stat. Soc. Ser. B 64, 583–639. doi: 10.1111/1467-9868.00353

[ref7001] Tenenbaum J. B. Griffiths T. L. Kemp C. (2006). Theory-based Bayesian models of inductive learning and reasoning. Trends in Cognitive Sciences. 10, 309–318. doi: 10.1016/j.tics.2006.05.009 16797219

[ref33] Tenenbaum J. B. Kemp C. Griffiths T. L. Goodman N. D. (2011). How to grow a mind: statistics, structure, and abstraction. Science 331, 1279–1285. doi: 10.1126/science.1192788, PMID: 21393536

[ref34] Tversky A. Kahneman D. (1974). Judgment under uncertainty: heuristics and biases. Science 185, 1124–1131. doi: 10.1126/science.185.4157.1124, PMID: 17835457

[ref35] Xu F. Garcia V. (2008). Intuitive statistics by 8-month-old infants. Proc. Natl. Acad. Sci. 105, 5012–5015. doi: 10.1073/pnas.0704450105, PMID: 18378901 PMC2278207

[ref36] Ye N. Namkoong H. (2024). Exchangeable Sequence Models Quantify Uncertainty Over Latent Concepts. arXiv preprint. arXiv:2408.03307.

[ref37] Zabell S. L. (1988). Symmetry and its discontents. In Skyrms B. Harper W. L., (Eds.), Causation, chance, and credence. (Vol. 1, 155–190). Dordrecht, Netherlands: Kluwer Academic Publishers.

[ref38] Zabell S. L. (1989). The rule of succession Erkenntnis 31 2–3 321 doi: 10.1007/BF01236567

[ref39] Zhang L. McCoy R. T. Sumers T. R. Zhu J. Q. Griffiths T. L. (2023). Deep de Finetti: recovering topic distributions from large language models. arXiv preprint. arXiv:2312.14226.

[ref40] Zhu J. Q. Sanborn A. N. Chater N. (2020). The Bayesian sampler: generic Bayesian inference causes incoherence in human probability judgments. Psychol. Rev. 127, 719–748. doi: 10.1037/rev0000190, PMID: 32191073 PMC7571263

